# Proteomic Changes of Glycolipid Pathways in Age-Related, Diabetic, and Post-Vitrectomy Cataracts

**DOI:** 10.3390/jcm13237287

**Published:** 2024-11-30

**Authors:** Christina Karakosta, Martina Samiotaki, George Panayotou, Dimitrios Papaconstantinou, Marilita M. Moschos

**Affiliations:** 1 School of Medicine, National and Kapodistrian University of Athens, 11527 Athens, Greece; 2 Biomedical Sciences Research Center “Alexander Fleming”, 16672 Vari, Greece; samiotaki@fleming.gr (M.S.); panayotou@fleming.gr (G.P.); 3 1st University Eye Clinic, “G. Gennimatas” General Hospital of Athens, National and Kapodistrian University of Athens, 11527 Athens, Greece; dpapaconstantinou@hotmail.com; 4 Department of Electrophysiology of Vision, 1st University Eye Clinic of Athens, 11527 Athens, Greece; moschosmarilita@yahoo.fr

**Keywords:** proteomics, lens, cataract, glycolipids, glycosphingolipid, age, diabetes, vitrectomy

## Abstract

**Background**: Alterations in glycolipid and glycosphingolipid pathways lead to compromised cell membranes and may be involved in cataract formation. However, the exact role of glycolipids in lens opacification is not completely understood. The aim of the current study is to investigate proteome complexity and the role of glycolipid and glycosphingolipid pathways in cataract formation. **Methods**: The anterior capsule and phacoemulsification (phaco) cassette contents were collected during cataract surgery from eleven participants with diabetic cataract (DC), twelve participants with age-related cataract (ARC), and seven participants with post-vitrectomy cataract (PVC). Liquid chromatography–mass spectrometry with data-independent acquisition (DIA) was used for the identification and quantification of proteins. **Results**: The results of this study revealed that the main significantly differentially expressed pathways in the ARC group compared to the DC and PVC groups in phaco cassette samples included the glycolipid metabolic, glycosphingolipid biosynthetic, and glycosphingolipid metabolic processes, with GLA being among the most significant proteins in the ARC group. Similarly, in the anterior capsule samples, the main significantly differentially expressed pathways in the ARC group compared to the DC and PVC groups were the glycolipid metabolic, glycosphingolipid biosynthetic, and glycosphingolipid metabolic processes, with ST3GAL5 being among the most significant proteins in the ARC group. **Conclusion**: Glycolipid and glycosphingolipid metabolic processes may be involved in cataract formation. ST3GAL5 may modify the cell-to-cell interaction induced by cell surface sugar chains, leading to the formation and progression of cataract. GLA, associated with the breakdown of glycolipids, may lead to cataract formation when a certain threshold is surpassed, secondary to increased glycolipid metabolism.

## 1. Introduction

Cataract is the main cause of reversible blindness [1,2]. In 2010, cataract contributed to more than a third of blindness cases and about one-fifth of visual impairments worldwide [3,4]. By 2025, about 50 million people are expected to have cataract [3], a fact that highlights the severity of this condition. To date, the treatment for cataract remains exclusively surgical, and even though phacoemulsification is the most common surgical procedure performed in developed countries, it requires sophisticated machinery and expertise [5]. The rapidly increasing number of cataract cases combined with the cost required for cataract surgery emphasize the importance of further research on cataract etiopathogenesis and the identification of non-invasive techniques for cataract treatment or even prevention.

The pathophysiology of cataract is multifactorial and it is not yet thoroughly comprehended. Known factors involved in cataract formation include aging, diabetes mellitus (DM), and previous ocular trauma, including any intraocular surgery and particularly vitrectomy [6]. The AREDS study showed that additional risk factors included sex, race, education, weight change, refraction, smoking, and multivitamin use [7]. The primary risk factor for all forms of cataract is aging, which underscores the complex nature of cataract development. This may be attributed to a combination of accumulated environmental damage, diminished lens repair mechanisms, and genetic susceptibility [7,8,9].

Glycolipids, which include glycosphingolipids, are essential components of cellular membranes, involved in various biological processes like cell signaling, membrane stability, and cellular stress responses [10,11]. Alterations in glycolipid and glycosphingolipid pathways lead to compromised cell membranes, and thus metabolic imbalances and cellular dysfunction may be involved in cataract formation [12].

Lens clarity is primarily determined by the integrity of the lens fiber cell plasma membranes. These structures have been the focus of extensive research on membrane lipids and proteins. Indeed, previous research has reported that abnormalities in membrane lipid composition are present in human cataracts and even small changes in lens membrane morphology may be enough to cause cataract [13,14]. Other studies have suggested that alterations in lipid metabolism, especially the high levels of triglycerides in aqueous humor, might be involved in cataractogenesis [15]. It has also been reported that the amount of sphingolipids increases with age and that these changes are exacerbated when age-related cataract is present [16]. Nevertheless, the exact role of glycolipids in lens opacification is not completely understood. Deciphering the role of these pathways could explore novel perspectives on the molecular level underlying cataractogenesis and detect potential non-interventional therapeutic targets to prevent or delay the onset of cataract [17].

The purpose of this research, which is a part of a larger project [4], is to investigate the role of glycolipid and glycosphingolipid pathways in age-related cataract formation, compared to other types of cataract, i.e., diabetic and post-vitrectomy cataract. By comprehensively examining alterations of these specific pathways in lenses with cataract, we aimed to uncover novel insights into the molecular mechanisms driving age-related lens opacification.

## 2. Materials and Methods

### 2.1. Human Subjects

This study was conducted according to the ethical principles of medical research involving human subjects according to the Declaration of Helsinki. All participants signed an informed consent form. The clinical data were analyzed and anonymized for patients’ confidentiality. Ethical approval (18534/20 July 2022) was granted by the institutional ethics board of the Hospital.

All patients presenting with visually significant cataract in the Ophthalmology Department were eligible for this study. All participants had visual acuity equal to or worse than 20/40 and they were organized into three distinct groups; diabetic cataract—DC, age-related cataract—ARC, and post-vitrectomy cataract—PVC. Group 1 (DC) included participants with diabetes mellitus (type 1 or 2) and aged younger than 65 years. Group 2 (ARC) included participants with no medical history of DM and aged older than 75 years. Group 3 (PVC) included participants of any age without a history of DM and a history of vitrectomy surgery for retinal detachment repair with gas tamponade in the past 12 months.

Participants with any previous ophthalmic trauma or chronic use of steroids, either via the topical or systemic route, were excluded from this study.

For every participant included in the study, their detailed medical history was recorded. In addition to this, a questionnaire was completed, which included the following: iris color, type of cataract, habits (alcohol or smoking), hours of sun exposure and the use of sunglasses, other medical problems (hypertension, thyroid disease, glaucoma, age-related macular degeneration), diet supplementary intake, and previous use of estrogens.

### 2.2. Sample Types and Collection

During cataract surgery, the following two sample types were collected from each participant: the anterior capsule and the content of the phacoemulsification (phaco) cassette. The anterior capsule samples consisted predominantly of lens epithelial cells. The phaco cassette samples, to a large extent, contained lens fiber cells. The majority of lens fiber cells were fully differentiated and organelles were lost.

**Figure 1:**
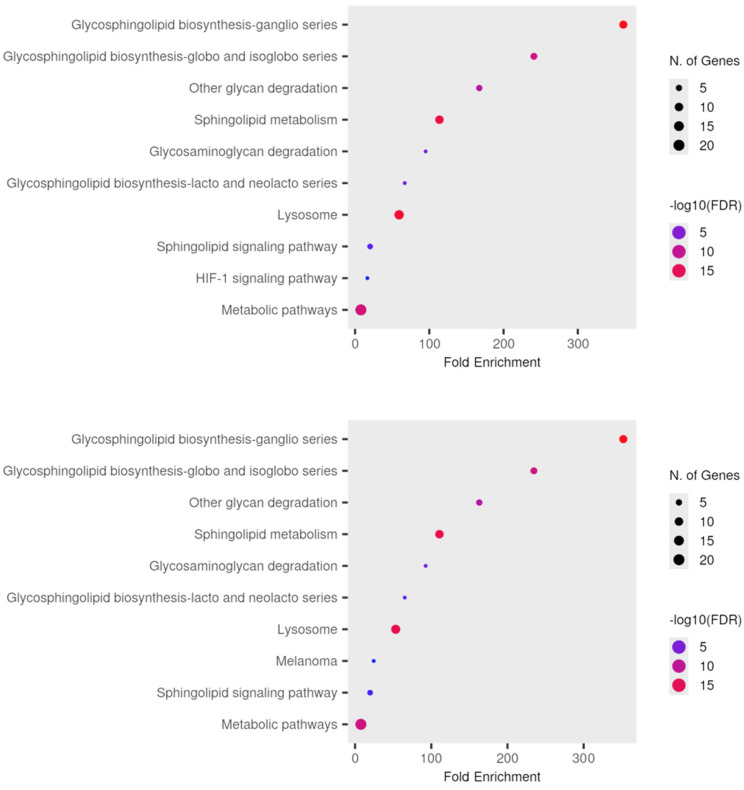
Dotplots of pathway enrichment analysis for proteins involved in glycolipid pathways that were significantly different in ARC samples when compared with the DC and ARC groups in the anterior capsule (**top**) and phaco cassette samples (**bottom**).

During cataract surgery, after the capsulorhexis was completed, the anterior capsule was collected using a Utrata forceps through the main incision, and was stored in a sterile plastic box containing 1–2 mL of Balanced Salt Solution (BSS). The time from the beginning of phacoemulsification till the end of the surgery was less than 5 min. At the end of the surgery, the content of the phaco cassette was collected from the phaco machine (Centurion^®^ Vision System, Alcon, Geneva, Switzerland). The phaco cassette contained BSS with the phacoemulsified pieces of crystalline lens along with the re-secreted aqueous humor, taking into consideration that the usual rate of aqueous humor production was 2.5 µL/min and the range of operation time was 5–10 min (Figure 1). All samples were immediately stored in a −80 °C freezer (Haier Biomedical, Qingdao, China). Dry ice was used for the transport of the samples to the lab.

### 2.3. Sample Preparation

Defrosting of all samples was conducted inside a pot full of ice at room temperature.

The anterior capsule samples were transferred to Eppendorf tubes (Sigma Aldrich, Darmstadt, Germany). The preparation of the anterior capsule sample began with the addition of 100 μL Lysis Buffer (4% SDS and 0.1 M DTT in 0.1 M TEAB) for cell lysis. Solutions were incubated at 99 °C for 10 min in AccuBlock™ Digital Dry Baths, Labnet and homogenized using Omni GLH international technology for several seconds. The tubes were then sonicated using a Bandelin Sonorex for 10 min. Then, the tubes were again transferred to the AccuBlock™ Digital Dry Baths and heated at 99 °C for 10 min. The heating and sonication were repeated twice. The next step of tryptic digestion was common for both sample types.

For the preparation of the phaco cassette samples, they were transferred from the cassette fluid bag containing BSS with the emulsified crystalline lens into Falcon tubes of 50 mL. In each falcon tube, 25 mL of the sample and an equal amount of 100% ethanol (=25 mL) were added. In each tube, 40 μL of beads (1:1 mixture of hydrophilic and hydrophobic SeraMag carboxylate-modified beads (Cytiva, Marlborough, MA, USA)) was added. Speedbead magnetic carboxylate-modified particles combine a fast magnetic response time and high binding capacity with a large surface area; high sensitivity, stability, and physical integrity; and fast reaction kinetics. The samples were mildly agitated for 30 min in order to let the proteins be captured by the beads. Phaco cassette fluid bags contained approximately 150–200 mL of fluid, and for condensation, the samples were subjected to centrifugation at 2200 rev/min (Sorvall RT7 PLUS, Waltham, MA, USA) for 10 min in order to collect the magnetic beads, and the supernatant was removed. Centrifugation was repeated. The samples were transferred to Eppendorf tubes and they were again centrifuged at 2000× *g* for 3 min. The suspension was removed from all tubes. The next step of tryptic digestion was common for both sample types.

The protein extracts from both sample types were processed by tryptic digestion using the Sp3 protocol, including a reduction (100 mM DTT) and an alkylation step in 200 mM iodoacetamide (Acros Organics, Thermo Fisher Scientific, Waltham, MA, USA). A total of 20 ug of beads (1:1 mixture of hydrophilic and hydrophobic SeraMag carboxylate-modified beads (Cytiva, Marlborough, MA, USA)) was added to each sample in the final proportion of 50% ethanol. A magnetic rack was used for the protein clean-up. The beads were washed twice with 80% ethanol and once with 100% acetonitrile (Fisher Chemical, Thermo Fisher Scientific, Waltham, MA, USA). Then, the digestion of the captured-on-beads proteins took place overnight at 37 °C under vigorous shaking (1200 rpm, Eppendorf Thermomixer, Hamburg, Germany) with 0.5 ug Trypsin/LysC (MS grade, Promega, Madison, WI, USA) prepared in 25 mM Ammonium bicarbonate. The next day, the peptides were purified by applying a modified Sp3 clean-up protocol, and finally solubilized in the mobile phase A (0.1% formic acid in water) and sonicated. The peptide concentration was determined through absorbance at 280 nm measurement using a nanodrop instrument. The samples were analyzed on a liquid chromatography–tandem mass spectrometry (LC-MS/MS) setup consisting of a Dionex Ultimate 3000 nanoRSLC coupled online with a Thermo Q Exactive HF-X Orbitrap mass spectrometer (Thermo Fisher Scientific, Waltham, MA, USA). The peptidic samples were directly injected and separated on a 25 cm long analytical C18 column (PepSep, 1.9 μm^3^ beads, 75 µm ID) using an one-hour-long run, starting with a gradient of 7% Buffer B (0.1% Formic acid in 80% Acetonitrile) to 35% for 40 min and followed by an increase to 45% in 5 min and a second increase to 99% in 0.5 min and then kept constant for equilibration for 14.5 min. A full MS was acquired in profile mode using a Q Exactive HF-X Hybrid Quadrupole-Orbitrap mass spectrometer (Thermo Fisher Scientific, Waltham, MA, USA), operating in the scan range of 375–1400 *m*/*z* using 120 K resolving power with an Automatic, Gain Control (AGC) of 3 × 10^6^ and maximum injection time (IT) of 60 ms followed by the data-independent acquisition method using 8 Th windows (a total of 39 loop counts) each with 15 K resolving power with an AGC of 3 × 10^5^ and max IT of 22 ms and normalized collision energy (NCE) of 26. Each sample was analyzed in three technical replicas.

### 2.4. Data Processing Protocol

Orbitrap raw data were analyzed in DIA-NN 1.8 (Data-Independent Acquisition by Neural Networks) through searching against the Human_Reviewed Proteome (downloaded from Uniprot, 50,516 protein entries, downloaded 11 April 2022) using the library free mode of the software, allowing up to two tryptic missed cleavages and a maximum of three variable modifications/peptide. Neural networks were used for peak selection. A spectral library was created from the DIA runs and used to reanalyze them (double search mode). The DIA-NN search was used with the oxidation of methionine residues, N-terminal methionine excision, and the acetylation of the protein N-termini set as variable modifications and the carbamidomethylation of cysteine residues as the fixed modification. The match-between-runs feature was used for all analyses and the output (precursor) was filtered at 0.01 FDR, and finally the protein inference was performed on the level of genes using only proteotypic peptides.

### 2.5. Statistical Analysis

Statistical analysis and data visualization were performed on Perseus Software (Version 1.6.15.0) (defined groups, *t*-test). The raw intensities were Log2-transformed, classified into the predefined clinical groups, and filtered based on valid values set to 50%. Missing values were handled by imputation based on normal distribution. Two sample *t*-tests were performed to compare the ARC-DC and ARC-PVC groups. The results of *t*-tests were visualized with volcano plots, with permutation-based FDR set at 0.05 and S0 (artificial within-group variance) set at 0.1. In addition, ANOVA tests were used for all group comparisons, and the results were visualized on heatmaps, with permutation-based FDR set at 0.05 and S0 (artificial within-group variance) set at 0.1. The basic characteristics (demographic data) of the patients were summarized with means and standard deviations (SD) for normally distributed continuous variables or medians and interquartile ranges (IQR) for skewed data. A two-way ANOVA (Analysis of Variance) test was performed to evaluate the effect of the categorical demographic factors on the values of the significant proteins for the three cataract groups. An ANCOVA test was performed to evaluate the effect of the qualitative demographic factors on the values of the significant proteins for the three cataract groups. A two-sided *p*-value of less than 0.05 was considered statistically significant. All analyses of demographic data were carried out using the R programming language and the RStudio IDE.

## 3. Results

### 3.1. Overview

#### 3.1.1. Demographics

Group 1 (DC) included 11 patients, group 2 (ARC) included 12 patients, and group 3 (PVC) included 7 patients. The three sample types were collected from all patients. In total, 16 women and 14 men were included. The average age of the participants was 67.1 ± 8.8 years old. The demographic data of the patients included in the study are presented in Table 1.

In total, 1639 proteins were identified in aqueous humor samples, 2815 proteins were detected in anterior capsule samples, and 2975 proteins were detected in phaco cassette content samples. Among all three sample types, 657 proteins were common and represent the core proteome of this study. Thirty proteins were common between the aqueous humor and phaco cassette content samples. A full list of the identified proteins is provided in the Supplementary Materials. Dotplots of gene enrichment analysis for the genes involved in glycolipid pathways are presented in Figure 1.

#### 3.1.2. Anterior Capsule Samples

**Figure 2:**
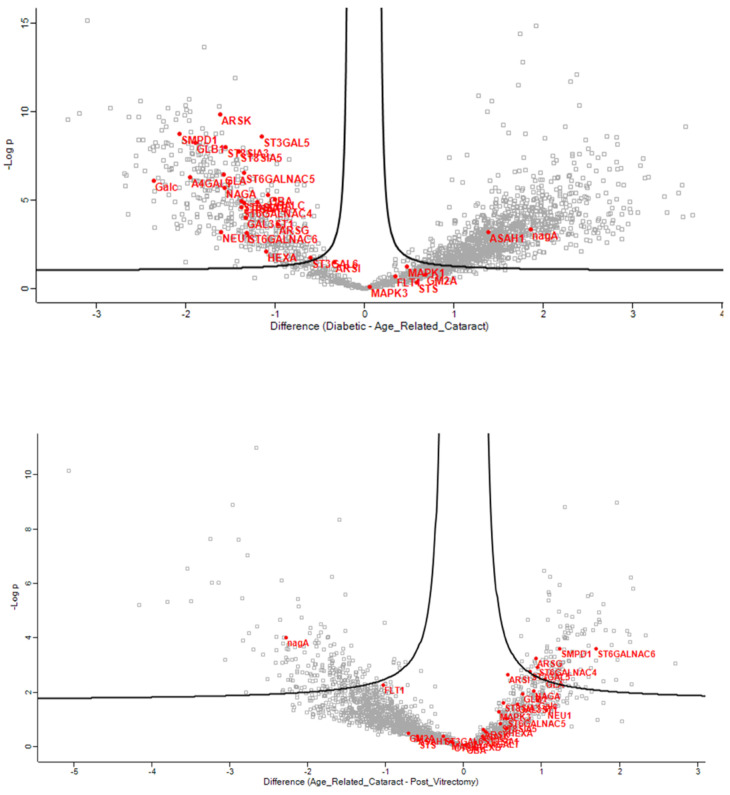
Volcano plots of the ARC-DC (**top**) and ARC-PVC group (**bottom**) comparison in anterior capsule samples showing proteins involved in the glycolipid metabolic process, which differed between the ARC and DC-PVC groups.

**Figure 3:**
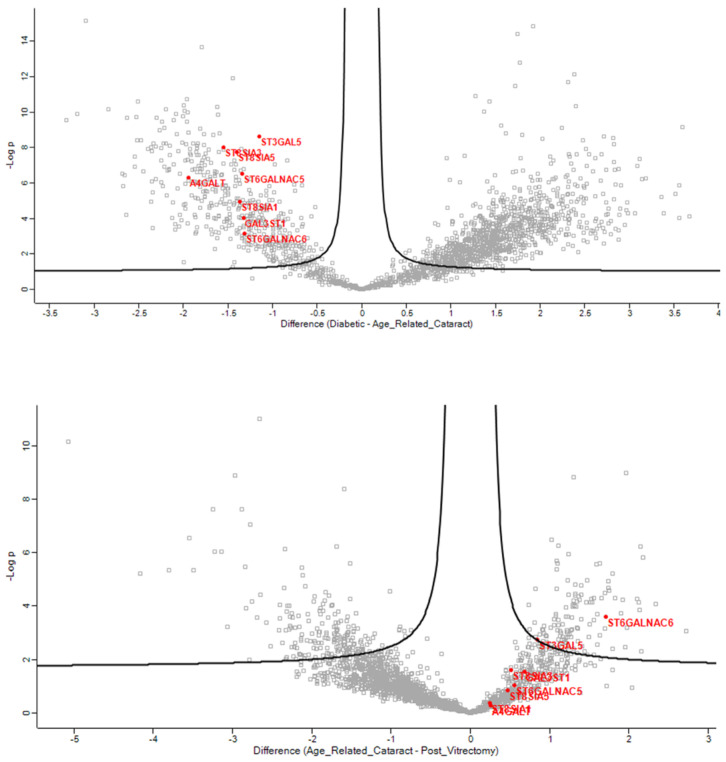
Volcano plots of the ARC-DC (**top**) and ARC-PVC group (**bottom**) comparison in anterior capsule samples showing the proteins involved in glycosphingolipid biosynthetic process, which differed between the ARC and DC-PVC groups.

**Figure 4:**
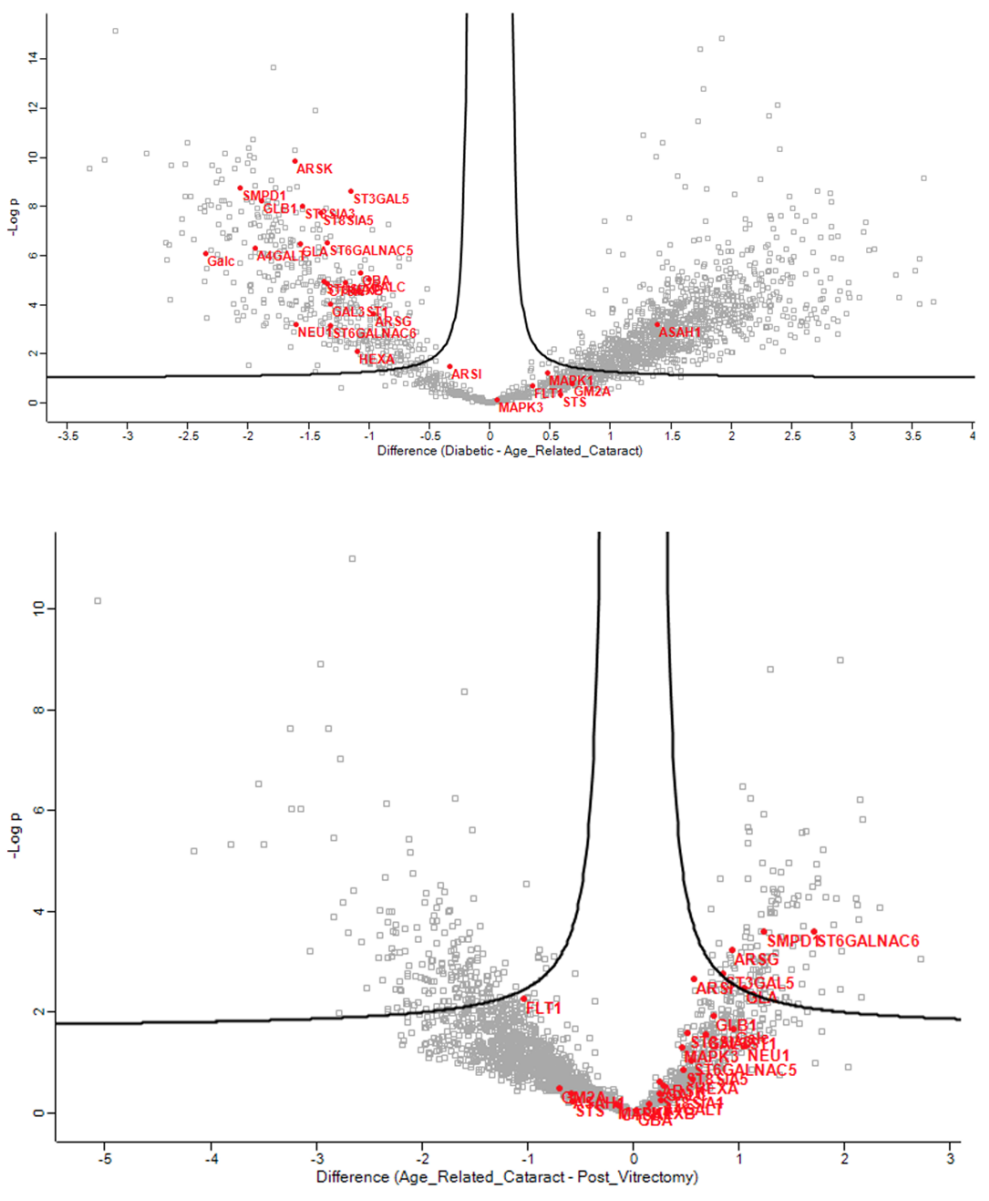
Volcano plots of the ARC-DC (**top**) and ARC-PVC group (**bottom**) comparison in anterior capsule samples showing the proteins involved in the glycosphingolipid metabolic process.

In the anterior capsule samples, the glycolipid metabolic (Figure 2), glycosphingolipid biosynthetic (Figure 3), and glycosphingolipid metabolic (Figure 4) process pathways were significantly impacted in ARC compared with both DC and PVC.

**Figure 5:**
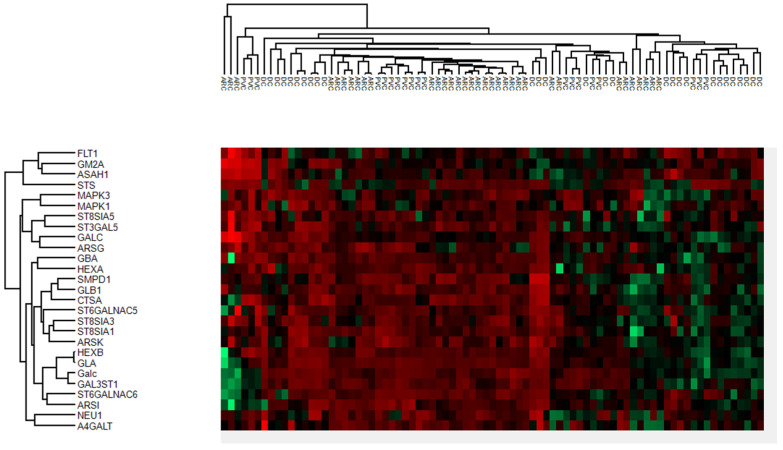
Heatmaps of anterior capsule samples showing the proteins involved in the glycosphingolipid metabolic and biosynthetic processes.

The proteins of anterior capsule samples involved in the glycosphingolipid metabolic and biosynthetic process pathways are shown in Figure 5.

**Figure 6:**
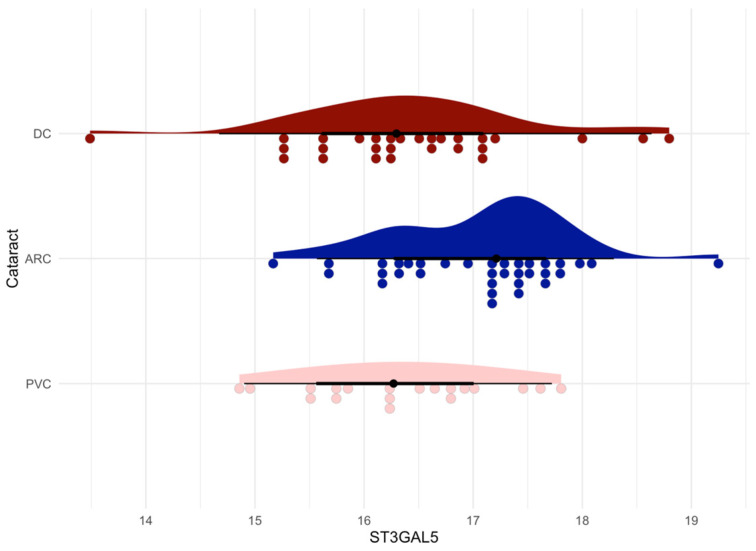
Raincloud plot of the ST3GAL5 protein (ARC-DC *p* = 0.0189, ARC-PVC *p* = 0.0204) in anterior capsule samples, showing that the levels of the protein are significantly different in the age-related group compared to the diabetic and post-vitrectomy groups.

The one-way ANOVA test showed that the ST3GAL5 protein was statistically significant in the ARC-DC (*p* = 0.0189) and ARC-PVC (*p* = 0.0204) comparisons of anterior capsule samples (Figure 6).

#### 3.1.3. Phaco Cassette Content Samples

**Figure 7:**
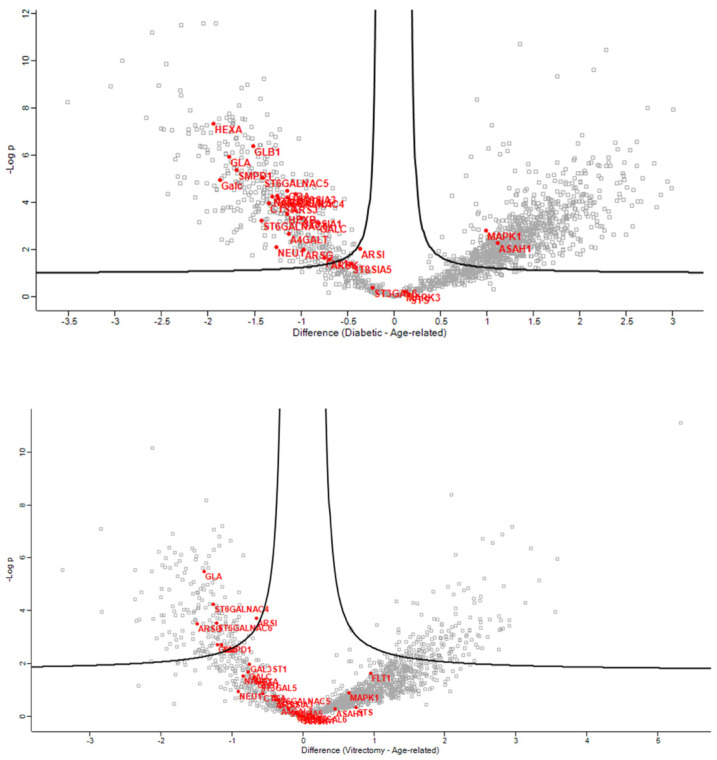
Volcano plots of the ARC-DC (**top**) and ARC-PVC group (**bottom**) comparison in phaco cassette content samples showing the proteins involved in the glycolipid metabolic process.

**Figure 8:**
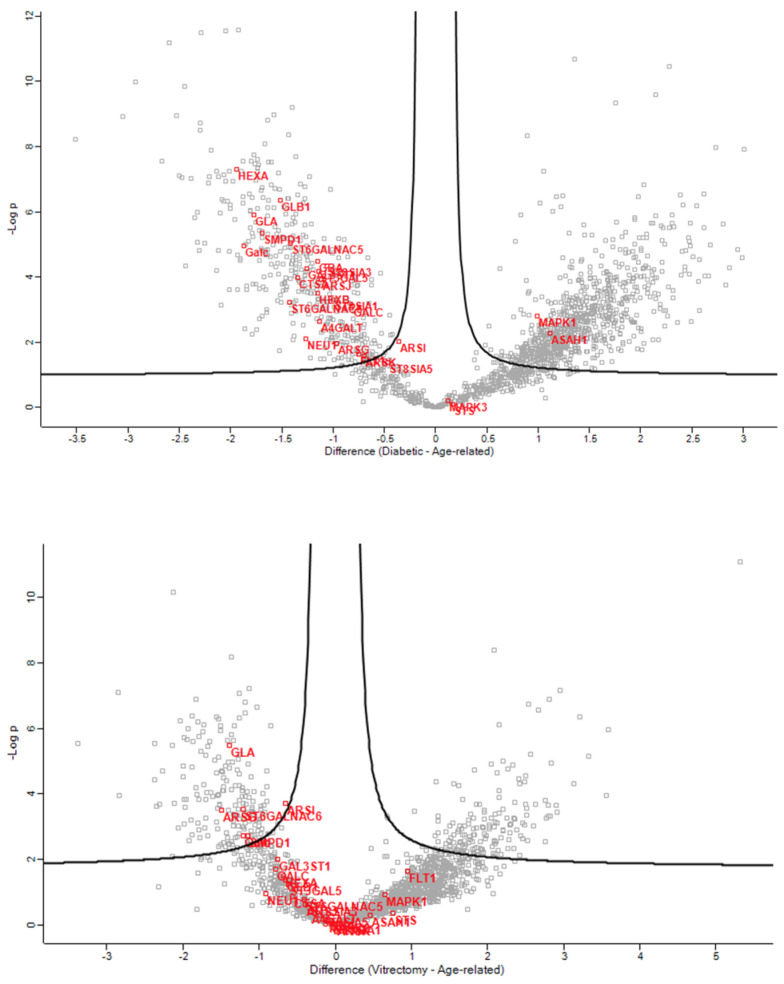
Volcano plots of the ARC-DC (**top**) and ARC-PVC group (**bottom**) comparison in phaco cassette content samples showing the proteins involved in the glycosphingolipid biosynthetic process.

**Figure 9:**
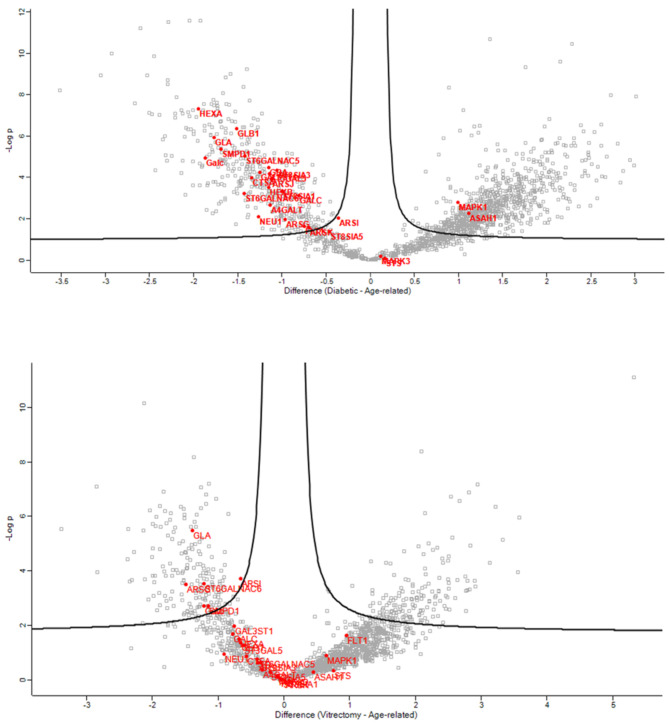
Volcano plots of the ARC-DC (**top**) and ARC-PVC group (**bottom**) comparison in phaco cassette content samples showing the proteins involved in the glycosphingolipid metabolic process.

In the phaco cassette content samples, the glycolipid metabolic (Figure 7), glycosphingolipid biosynthetic (Figure 8), glycosphingolipid metabolic (Figure 9), and glycosylceramide metabolic process pathways were significantly impacted in ARC compared with both DC and PVC.

**Figure 10:**
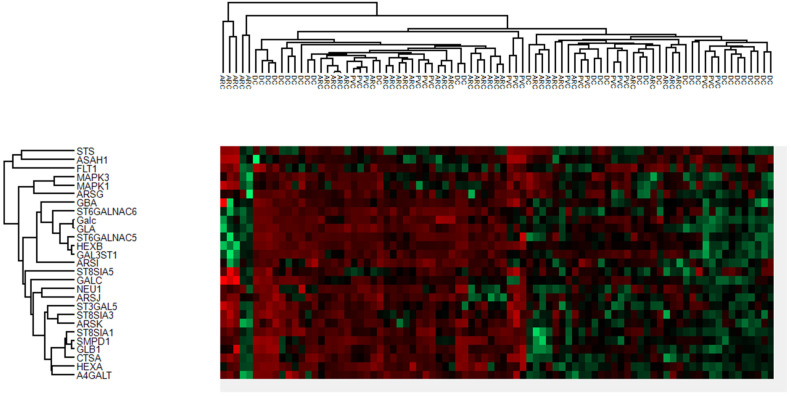
Heatmaps of the phaco cassette content samples showing the proteins involved in glycosphingolipid metabolic and biosynthetic processes.

These pathways were not significant in the aqueous humor samples, suggesting that these differences reflect changes in the lens. The proteins of the phaco cassette content samples involved in the glycosphingolipid metabolic and biosynthetic process pathways are shown in the heatmap in Figure 10.

**Figure 11:**
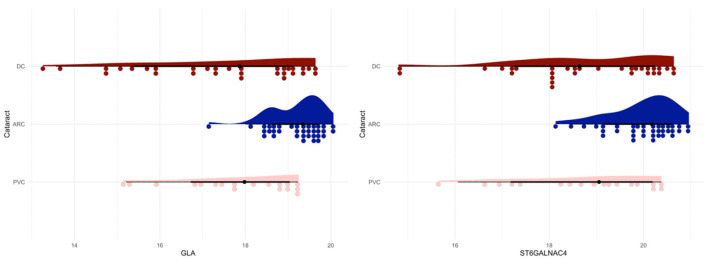
Raincloud plots of GLA protein (**left**) (ARC-DC *p* < 0.0001, ARC-PVC *p* < 0.01) and ST6GALNAC4 protein (**right**) (ARC-DC *p* < 0.001, ARC-PVC *p* = 0.01) in the phaco cassette content samples that show that the levels of both proteins are significantly different in the age-related group compared to the diabetic and post-vitrectomy groups.

The one-way ANOVA test showed that the GLA protein was statistically significant in ARC-DC (*p* = 0.00000223) and ARC-PVC (*p* = 0.00172) comparisons in the phaco cassette content samples (Figure 11).

Moreover, the ST6GALNAC4 protein was statistically significant in the ARC-DC (*p* = 0.000306) and ARC-PVC (*p* = 0.00214) comparisons in the phaco cassette content samples (Figure 10).

### 3.2. Demographic Data

Two-way ANOVA and ANCOVA tests were performed to evaluate the effect of the demographic data of the patients on the most significant proteins of each group. These included the ST3GAL5 protein in anterior capsule samples and the GLA and ST6GALNAC4 proteins in the phaco cassette content samples. The tests revealed no statistically significant effect (*p* > 0.05) on the protein values of the three groups for the following factors: sex, height, weight, axial length, smoking status, aspirin intake, and the use of diet supplements. It is important to note that this analysis was based on subgroup data, with relatively small sample sizes in each group. Consequently, drawing definitive conclusions may not be appropriate due to the limitation in sample size.

## 4. Discussion

The results of this study revealed that the main significantly differentially expressed pathways in the ARC group compared to the DC and PVC groups in the phaco cassette samples included the glycolipid metabolic, glycosphingolipid biosynthetic, and glycosphingolipid metabolic processes, with GLA and ST6GALNAC4 being among the most significant proteins in the ARC group.

Similarly, in the anterior capsule samples, the main significantly differentially expressed pathways in the ARC group compared to the DC and PVC groups were the glycolipid metabolic, glycosphingolipid biosynthetic, and glycosphingolipid metabolic processes, with ST3GAL5 being among the most significant proteins in the ARC group.

Previous studies showed that ordered lipids scatter more light than disordered lipids and that increased light scattering in cataractous lenses may be due to an increase in the lipid order of lens membranes [18]. Glycosphingolipids comprise principal elements of cell membranes and are involved in cellular differentiation and interaction as second messengers [19]. Previous studies have investigated the role of sphingolipids in the transparency of human lenses. The relative content of sphingolipids in human lenses is estimated to be more than 60%, and Borchman et al. suggested that humans have adapted with a higher sphingolipid content in their lens membranes compared to other species in order to resist oxidation [20]. This resistance to oxidation would allow human lens cell membranes to remain clear for longer, thus prolonging lens transparency [14,20]. However, increased sphingolipids in aged and cataractous lenses may indicate phospholipid oxidation, which is involved in lens opacification [14]. Huang et al. showed that the relative and absolute amounts of sphingolipids increased with age, and that these changes were exacerbated by the presence of cataract [16]. Ceramide is the key regulator of the sphingolipid metabolic process and is essential for lipid signaling. Stress causes increased cellular levels of ceramide which in turn causes apoptosis and cell death [21]. In the aging lens, concentrations of ceramides are increased, and their role in the development of ARC may be explained by the increased apoptosis in lens epithelial cells [22,23,24,25]. In the present study, the glycosylceramide metabolic process was significantly different in the ARC group compared to the DC and PVC groups, suggesting a possible involvement of glycosphingolipids and ceramide in the formation of ARC.

Glycolipids, present in the outer parts of cell membranes, make up less than 1% of the total human lens lipid, but their role is essential for both lens differentiation and the maturation of lens epithelial cells into lens fibers [14]. Gangliosides are complex acidic glycolipids and, in particular, ganglioside GM3 synthase (ST3GAL5) is a primary glycosyltransferase for the synthesis of complex gangliosides. It has been reported that the human lens accumulates gangliosides (such as GM3, GM2, and GM1) and that those age-related changes in lens glycolipids may alter the intercell interaction induced by cell surface sugar chains, leading to lens opacification [25,26,27]. In the present study, ST3GAL5 was significant in the anterior capsule samples of the ARC group compared to both the DC and PVC groups, and it could be hypothesized that they are particularly implicated in cataract formation by modifying cell membranes and cell-to-cell interactions. In particular, altered proteoforms of ST3GAL5, which is responsible for the formation of GM3 ganglioside, may affect its activity, leading to the disrupted synthesis of gangliosides including GM3 and may impair lens fiber cell membrane integrity and intercellular communication, contributing to lens opacification.

ST6GALNAC4 is a type II membrane protein that catalyzes the transfer of sialic acid from CMP–sialic acid to galactose-containing substrates and it prefers glycoproteins rather than glycolipids as substrates [28,29]. It was found to be significant in the ARC group compared to the DC and PVC groups in phaco cassette samples. GLA is associated with the breakdown of glycolipids, preventing their accumulation in the lens, thus maintaining lens transparency. In the present study, GLA was significantly different in the ARC group compared to the DC and PVC groups in phaco cassette content samples. An interpretation of these results would suggest a possible increased activity secondary to increased glycolipid metabolism. A hypothesis would be that with age, once a certain threshold of GLA activity is surpassed, lens opacification and ARC may occur, likely secondary to increased glycolipid metabolism. Another hypothesis would be that elevated GLA activity may lead to the excessive breakdown of glycolipids, resulting in the accumulation of metabolic byproducts such as ceramides or other sphingolipid intermediates in the lens. Regarding ST3GAL5, which is responsible for the formation of GM3 ganglioside, altered proteoforms of it could affect its activity, leading to disrupted synthesis of gangliosides, including GM3, and may impair lens fiber cell membrane integrity and intercellular communication, contributing to lens opacification. The changes in protein species related to glycolipid and glycosphingolipid pathways may highlight the proteome’s dynamic shifts during cataract progression and support the hypothesis that these alterations contribute to the structural and functional changes leading to ARC. The complexity of the proteome in the lens, in relation to these glycolipid-related processes, suggests that the diversity in proteoforms and protein species may play a crucial role in mediating the pathological changes associated with ARC. The intricate proteome complexity in the lens underscores the need to investigate not just individual protein species but their interactions and modifications during aging and cataract development.

The current study should be interpreted within the context of its limitations. An age-matched transparent lens group was not included in the current study and it would be valuable for distinguishing the proteins/pathways involved in cataract formation and those that are simply due to the aging process. Clear lens extraction in those patients for refractive reasons could be used as a baseline, and further elucidate the mechanisms which are exclusively involved in age-related cataract formation. The sample size of the current study is relatively small and larger studies with matched cohorts regarding the demographic differences among the cataract groups would be quite useful to validate the present results. Moreover, in this study, all cataract types were combined in order to provide an overall investigation of cataract formation pathways. Using subgroups for the types of cataract may provide precious data regarding the different mechanisms that cause each distinct cataract type. Nevertheless, in this study, three different sample types were collected from each patient and this may serve as a control, taking into account that the up-/down-regulation of pathways/proteins appears to follow a consistent pattern across all three sample types. In this study, phaco cassette samples were collected after phacoemulsification surgery. Even though phacoemulsification may generate free radical-mediated oxidative stress, studying such samples may open new horizons regarding the development of a method that focuses on cataract investigation. Finally, it should be highlighted that data-independent acquisition (DIA) provides broad proteomic coverage, but it may lack the sensitivity to detect low-abundance proteins.

## 5. Conclusions

Overall, this study has generated a comprehensive and rich atlas of the lens proteome, revealing key insights into the molecular mechanisms underlying cataract formation. The glycolipid and glycosphingolipid metabolic processes appear to be involved in cataract formation. The proteome complexity in these pathways suggests that changes in protein abundancy, such as ST3GAL5 and GLA, related to glycolipid metabolism may play a crucial role in these alterations. These findings highlight the possible dynamic shifts in proteoforms and protein species in the lens during cataract progression, further underscoring the complex interactions within the proteome that drive the pathogenesis of age-related cataracts. Future studies with larger sample sizes, which validate protein abundance and tissue localization, would strengthen the results of the current study. The results of the present study provide insight into potential pharmacotherapeutic targets. Implications for future research and clinical applications may include alternative treatment of age-related cataract such as solid lipid nanoparticles to target certain proteins, like GLA and ST3GAL5, in order to slow down or even prevent age-related cataract progression.

## Figures and Tables

**Table 1:** Cohort composition.

	Group 1 (DC)	Group 2 (ARC)	Group 3 (PVC)
Subjects	11	12	7
Mean age (years, mean ± SD)	61.7 ± 4.3	79.6 ± 4.2	60 ± 10.2
Sex (male/female)	7:4	5:7	2:5
Mean height (cm, mean ± SD)	171.45 ± 7.13	164.08 ± 9.98	168.88 ± 7.08
Mean weight (kg, mean ± SD)	94.55 ± 6.82	70.58 ± 13.75	77.88 ± 13.35
Mean AL (mm, mean ± SD)	23.10 ± 0.80	23.69 ± 0.97	24.52 ± 1.49
Smoking (Yes/No)	4:7	3:9	0:7
Aspirin intake (Yes/No)	1:10	1:11	0:7
Diet supplementary intake (Yes/No)	3:8	5:7	1:6

SD = standard deviation; AL = axial length.

## Data Availability

The mass spectrometry proteomics data have been deposited in the ProteomeXchange Consortium via the PRIDE partner repository with the dataset identifiers PXD045547, PXD045554, and PXD045557 [30].

## Supplementary Materials

The following supporting information can be downloaded at: https://www.mdpi.com/article/10.3390/jcm13237287/s1.

## References

[1] Javitt J.C., Wang F., West S.K. (1996). Blindness Due to Cataract: Epidemiology and Prevention. Annu. Rev. Public Health.

[2] Delcourt C., Carrière I., Delage M., Descomps B., Cristol J.P., Papoz L. (2003). Associations of Cataract with Antioxidant Enzymes and Other Risk Factors: The French Age-Related Eye Diseases (POLA) Prospective Study. Ophthalmology.

[3] Khairallah M., Kahloun R., Bourne R., Limburg H., Flaxman S.R., Jonas J.B., Keeffe J., Leasher J., Naidoo K., Pesudovs K. (2015). Number of People Blind or Visually Impaired by Cataract Worldwide and in World Regions, 1990 to 2010. Investig. Ophthalmol. Vis. Sci..

[4] Karakosta C., Samiotaki M., Panayotou G., Papakonstantinou D., Moschos M.M. (2024). Role of Actin-Binding Proteins in Cataract Formation. Cytoskeleton.

[5] Allen D., Vasavada A. (2006). Cataract and Surgery for Cataract. BMJ Br. Med. J..

[6] Karakosta C., Samiotaki M., Panayotou G., Papaconstantinou D.S., Moschos M.M., Karakosta C., Samiotaki M., Panayotou G., Papaconstantinou D.S., Moschos M.M. (2024). Lens Cytoskeleton: An Update on the Etiopathogenesis of Human Cataracts. Cureus.

[7] Chang J.R., Koo E., Agrón E., Hallak J., Clemons T., Azar D., Sperduto R.D., Ferris F.L., Chew E.Y. (2011). Age-Related Eye Disease Study Group Risk Factors Associated with Incident Cataracts and Cataract Surgery in the Age-Related Eye Disease Study (AREDS). Ophthalmology.

[8] Hennis A., Wu S.Y., Nemesure B., Leske M.C. (2004). Risk Factors for Incident Cortical and Posterior Subcapsular Lens Opacities in the Barbados Eye Studies. Arch. Ophthalmol..

[9] Kanthan G.L., Wang J.J., Rochtchina E., Tan A.G., Lee A., Chia E.M., Mitchell P. (2008). Ten-Year Incidence of Age-Related Cataract and Cataract Surgery in an Older Australian Population. The Blue Mountains Eye Study. Ophthalmology.

[10] Bogdanov M., Mileykovskaya E., Dowhan W. (2008). Lipids in the Assembly of Membrane Proteins and Organization of Protein Supercomplexes: Implications for Lipid-Linked Disorders. Subcell. Biochem..

[11] Hunter C.D., Guo T., Daskhan G., Richards M.R., Cairo C.W. (2018). Synthetic Strategies for Modified Glycosphingolipids and Their Design as Probes. Chem. Rev..

[12] Cabrera-Reyes F., Parra-Ruiz C., Yuseff M.I., Zanlungo S. (2021). Alterations in Lysosome Homeostasis in Lipid-Related Disorders: Impact on Metabolic Tissues and Immune Cells. Front. Cell Dev. Biol..

[13] Gilliland K.O., Freel C.D., Johnsen S., Craig Fowler W., Costello M.J. (2004). Distribution, Spherical Structure and Predicted Mie Scattering of Multilamellar Bodies in Human Age-Related Nuclear Cataracts. Exp. Eye Res..

[14] Borchman D., Yappert M.C. (2010). Lipids and the Ocular Lens. J. Lipid Res..

[15] Wang J., Zhang Y., Li W., Zhou F., Li J. (2022). Changes in the Lipid Profile of Aqueous Humor From Diabetic Cataract Patients. Transl. Vis. Sci. Technol..

[16] Huang L., Grami V., Marrero Y., Tang D., Yappert M.C., Rasi V., Borchman D. (2005). Human Lens Phospholipid Changes with Age and Cataract. Investig. Ophthalmol. Vis. Sci..

[17] Home Page|Cell Communication and Signaling. https://biosignaling.biomedcentral.com/.

[18] Borchman D. (2021). Lipid Conformational Order and the Etiology of Cataract and Dry Eye. J. Lipid Res..

[19] Azizov S., Sharipov M., Lim J.M., Tawfik S.M., Kattaev N., Lee Y.I. (2021). Solvent-Resistant Microfluidic Paper-Based Analytical Device/Spray Mass Spectrometry for Quantitative Analysis of C18 -Ceramide Biomarker. J. Mass. Spectrom..

[20] Borchman D., Yappert M.C., Afzal M. (2004). Lens Lipids and Maximum Lifespan. Exp. Eye Res..

[21] Stith J.L., Velazquez F.N., Obeid L.M. (2019). Advances in Determining Signaling Mechanisms of Ceramide and Role in Disease. J. Lipid Res..

[22] Hannun Y.A., Obeid L.M. (2018). Sphingolipids and Their Metabolism in Physiology and Disease. Nat. Rev. Mol. Cell Biol..

[23] Woodcock J. (2006). Sphingosine and Ceramide Signalling in Apoptosis. IUBMB Life.

[24] Young M.M., Wang H.G. (2018). Sphingolipids as Regulators of Autophagy and Endocytic Trafficking. Adv. Cancer Res..

[25] Cavallotti C.A.P., Cerulli L. (2008). Age-Related Changes of the Human Eye. Age-Related Changes of the Human Eye.

[26] Ogiso M., Komoto M., Okinaga T., Koyota S., Hoshi M. (1995). Age-Related Changes in Ganglioside Composition in Human Lens. Exp. Eye Res..

[27] Cavdarli S., Groux-Degroote S., Delannoy P. (2019). Gangliosides: The Double-Edge Sword of Neuro-Ectodermal Derived Tumors. Biomolecules.

[28] Hand-Picked Antibodies, ELISA Kits & Proteins for Research. https://www.antibodies-online.com/.

[29] ST6GALNAC4 ST6 N-Acetylgalactosaminide Alpha-2,6-Sialyltransferase 4 [Homo Sapiens (Human)]-Gene-NCBI. https://www.ncbi.nlm.nih.gov/gene/27090.

[30] Deutsch E.W., Bandeira N., Perez-Riverol Y., Sharma V., Carver J.J., Mendoza L., Kundu D.J., Wang S., Bandla C., Kamatchinathan S. (2023). The ProteomeXchange Consortium at 10 Years: 2023 Update. Nucleic Acids Res..

